# Characterising the premonitory stage of migraine in children: a clinic-based study of 100 patients in a specialist headache service

**DOI:** 10.1186/s10194-016-0689-7

**Published:** 2016-10-21

**Authors:** N. Karsan, P. Prabhakar, P. J. Goadsby

**Affiliations:** 1 Headache Group, Department of Basic and Clinical Neuroscience, Institute of Psychiatry, Psychology and Neuroscience, King’s College London, London, UK; 2 Department of Paediatric Neurology, Great Ormond Hospital for Children NHS Foundation Trust, London, UK; 3 NIHR-Wellcome Trust King’s Clinical Research Facility, King’s College Hospital, London, SE5 9PJ UK

**Keywords:** Migraine, Premonitory, Paediatrics

## Abstract

**Background:**

The premonitory stage of migraine attacks, when symptomatology outside of pain can manifest hours to days before the onset of the headache, is well recognised. Such symptoms have been reported in adults in a number of studies, and have value in predicting an impending headache. These symptoms have not been extensively studied in children. We aimed to characterise which, if any, of these symptoms are reported in children seen within a Specialist Headache Service.

**Methods:**

We reviewed clinic letters from the initial consultation of children and adolescents seen within the Specialist Headache Service at Great Ormond Street Hospital between 1999 and 2015 with migraine in whom we had prospectively assessed clinical phenotype data. We randomly selected 100 cases with at least one premonitory symptom recorded in the letter. For these patients, the age at headache onset, presence of family history of headache, headache diagnosis, presence of episodic syndromes which may be associated with headache, developmental milestones, gestation at birth, mode of delivery and presence of premonitory symptoms occurring before or during headache were recorded.

**Results:**

Of the 100 patients selected, 65 % were female. The age range of the patients was 18 months to 15 years at the time of headache onset. The most common diagnosis was chronic migraine in 58 %, followed by episodic migraine (29 %), New Daily Persistent Headache with migrainous features (8 %) and hemiplegic migraine (5 %). A history of infantile colic was noted in 31 % and was the most common childhood episodic syndrome associated with migraine. The most common premonitory symptoms recorded were fatigue, mood change and neck stiffness. The commonest number of reported premonitory symptoms was two.

**Conclusion:**

Premonitory symptoms associated with migraine are reported in children as young as 18 months, with an overall clinical phenotype comparable to adults. Better documentation of this stage will aid parents and clinicians to better understand the phenotype of attacks, better recognise migraine and thus initiate appropriate management. Larger studies with a broader base are warranted to understand the extent and implications of these symptoms for childhood and adolescent migraine.

## Background

Migraine is a complex and disabling pain condition and affects 4–10 % of school aged children [1, 2]. Migraine is characterised by recurrent episodes of throbbing head pain, accompanied by other symptoms including nausea, vomiting and sensory sensitivities [3]. Additionally, it has been increasingly well recognised that there are other significant and disabling symptoms associated with migraine and these can include but are not limited to cognitive change, mood disturbance, neck discomfort and fatigue [3]. These premonitory symptoms can occur hours to days before the onset of pain, can occur or persist in the headache phase, and can indeed persist after the headache has resolved in the postdrome [4]. Various studies have documented different prevalence’s and phentoypes of premonitory symptoms in adults [5–11], while less is understood about this phase in childhood migraine.

The phenotype of migraine differs between adults and children [12] and this has been attributed to changes in brain development and myelination [13]. It is also well recognised that syndromes where pain is not a prominent feature can be precursors to migraine in young children; these are classified as ‘episodic syndromes that may be associated with migraine’ in the ICHD-3 beta classification of headache disorders [3]. While the episodic disorders associated with paediatric migraine have been described over some time [14–17], the difficulties in obtaining accurate histories from small children, and a generally lessened interest in premonitory symptoms, apart from one study in a clinic population [18], have resulted in little data in the literature. Given the increase in research interest into the premonitory stage of migraine attacks [5–7, 19], the biology of premonitory-like symptomatology and its contribution to understanding how to advance migraine therapeutics [20], it is timely to address these symptoms in childhood migraine. Clarifying the symptoms may help parents understand what their child is experiencing, and clinicians relate to, and explain these symptoms, to families. Here we aimed to examine the phenotype of premonitory symptoms and premonitory symptoms occurring during headache for 100 children seen within a Specialist Headache Service at Great Ormond Street Hospital for Children in London.

## Methods

The study population comprised children and adolescents seen within the Specialist Headache Clinic at Great Ormond Street Hospital for Children between 1999 and 2015 by authors (PP and PJG). All the patients had a final diagnosis of migraine or New Daily Persistent Headache with migrainous features. All the patients had been seen in the presence of one or more parent or guardian and where possible the patient histories were taken from the patient themselves. The very first consultation in this clinic is where a detailed headache history is taken and documented. Clinic letters from the initial headache consultation of children seen were examined for data acquisition.

Patients (*n* = 100) for which the clinic letters documented premonitory symptomatology or premonitory symptoms occurring during headache were randomly selected for this study. Typical migraine aura and cranial autonomic symptoms were excluded. The data were compiled and analysed with the objective of carrying out a service evaluation, which in UK practice does not require Research Ethics Committee review (http://www.hra-decisiontools.org.uk/research/).

### Letter structure

Prospectively, for each patient as part of the routine clinical assessment, the age of headache onset, headache diagnosis, presence of episodic syndromes that may be associated with migraine, age at meeting developmental milestones of walking and talking, gestation at birth and mode of delivery, presence of positive family history of headache and the presence of various premonitory symptoms were noted. The premonitory symptoms for which information was collected were all those reported in any letter.

### Data collation

The number of premonitory symptoms reported for each patient was recorded and the associations with age, sex and headache diagnosis were analysed. Additionally, the relationship between episodic syndromes that may be associated with migraine and particular similar premonitory symptoms was analysed.

### Data analysis

Symptoms were tabulated in Excel to provide summary data. Qualitative differences were examined using the Chi-squared test. Analyses were performed using IBM SPSS Statistics 22. *P* < 0.05 was considered significant.

## Results

Data collection was made for patients seen between 1999 and 2015. Patients (*n* = 100) seen within this period, who had at least one premonitory symptom recorded in the initial consultation letter, were randomly selected. All of these patients fulfilled ICHD-3 beta criteria for the diagnosis of episodic migraine, chronic migraine, hemiplegic migraine or New Daily Persistent Headache with migrainous features [3].

### Demographics and diagnoses

The mean age of subjects at symptom onset was 8 years (range 1.5–15 years). The commonest diagnosis was chronic migraine in 58 %, followed by episodic migraine in 29 %, New Daily Persistent Headache with migrainous features in 8 %, sporadic hemiplegic migraine in 4 % and familial hemiplegic migraine in 1 %. Of patients, 65 % were female and 81 % of all patients had a positive family history of headache. Most of the cohort were born by spontaneous vaginal delivery (73 %), with the vast majority (80 %) being born within 37 to 42 weeks’ gestation.

### Premonitory features defined

**Fig. 1:**
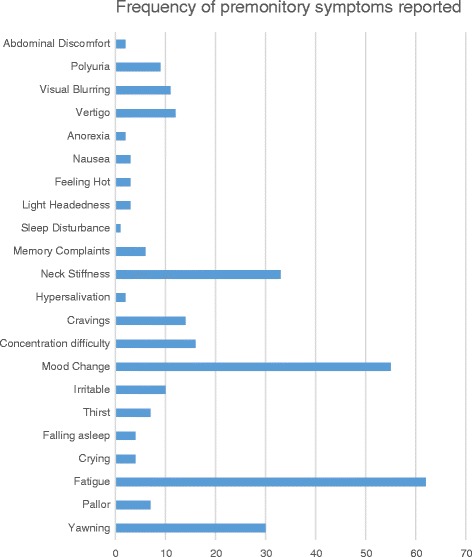
Frequency of different premonitory symptoms reported

Premonitory symptoms were defined as symptoms recognised as occurring prior to the onset of pain and any non-migraine defining features occurring during the pain. These included: vertigo, neck stiffness, visual blurring and light headedness but excluded photophobia, phonophobia, osmophobia and nausea. We also excluded any cranial autonomic features because of their discrete pathophysiology [21]. Typical migraine aura symptoms were also excluded. Data were collected for any symptom within these criteria and twenty-two symptoms were included in the final dataset (Fig. 1).

### Occurrence of premonitory features

**Fig. 2:**
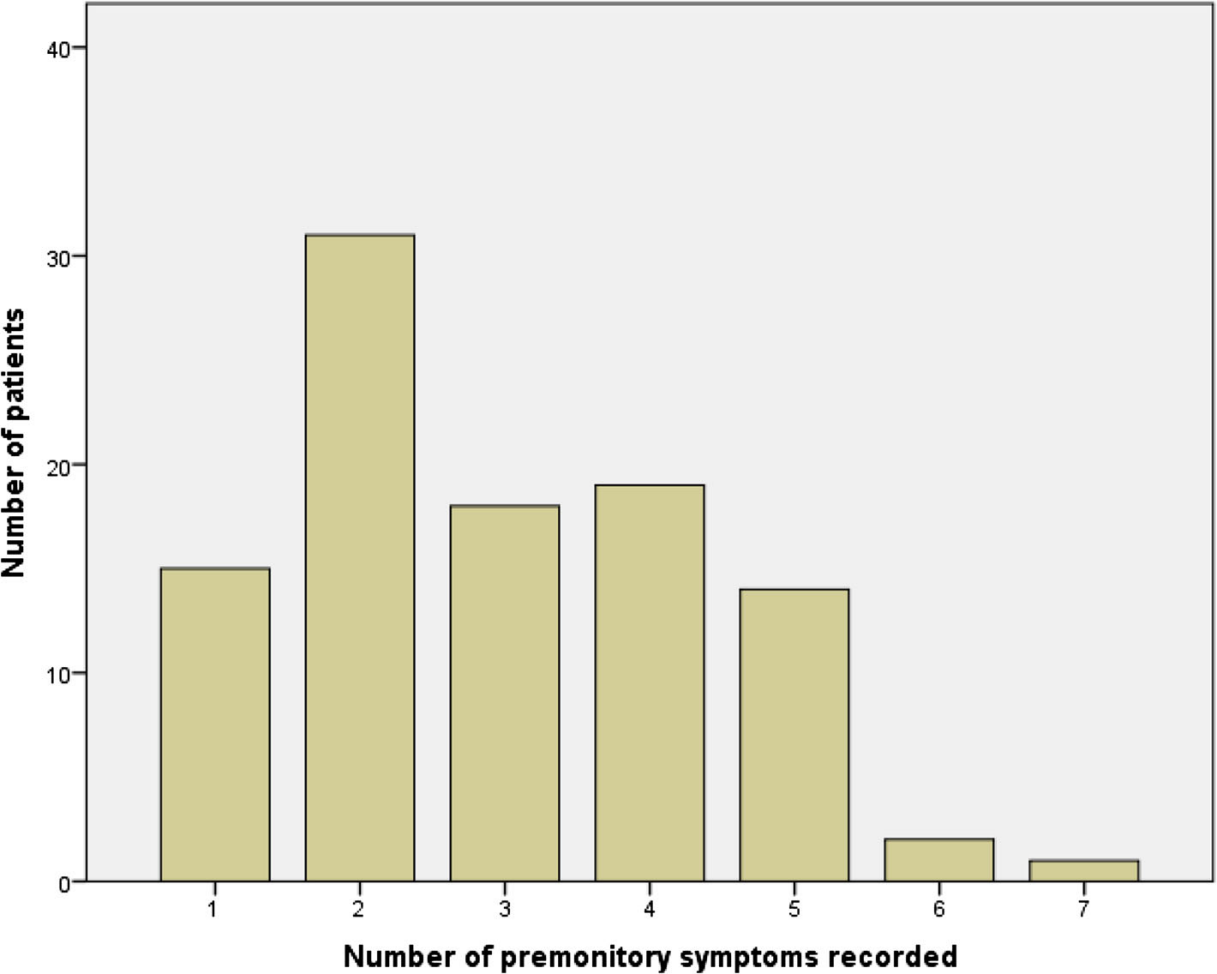
Number of premonitory symptoms reported by number of patients

**Fig. 3:**
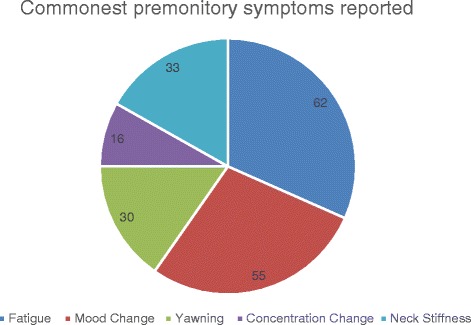
Number of patients reporting the 5 most common premonitory symptoms

Two premonitory symptoms was the commonest number of premonitory symptoms and was reported by 31 % of patients (Fig. 2). The mean number of premonitory symptoms reported was three. Two or more premonitory symptoms were reported by 85 % of patients. The most common premonitory symptoms reported were fatigue, mood change, yawning, concentration difficulty and neck stiffness (Fig. 3). There was no significant association with the development of two or more premonitory symptoms and age of symptom onset, gender, or with headache diagnosis (Table 1).

**Table 1:** Analysis of association between those patients reporting at least two premonitory symptoms and sex, age of onset of symptoms and headache diagnosis

	Subgroups	*N*	At least 2 premonitory symptoms	*P* value
Sex	Male	35	28	0.38
	Female	65	58	
Age of onset of symptoms (years)	<5	14	11	0.83
	5–10	39	33	
	10–12	28	25	
	>12	19	16	
Headache diagnosis	Episodic migraine	28	20	0.079
	Chronic migraine	59	52	
	Hemiplegic migraine (familial or sporadic)	5	5	
	New daily persistent headache with migrainous features	8	8	

### Association of premonitory symptoms with episodic symptoms

**Fig. 4:**
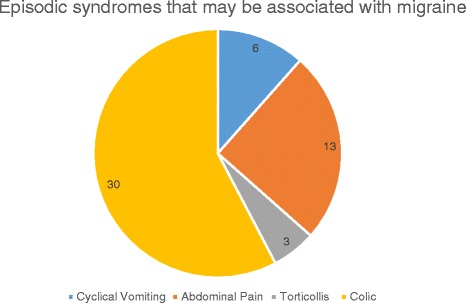
Episodic syndromes that may be associated with migraine reported in the study population

The commonest reported episodic syndrome that may be associated with migraine was infantile colic in 31 %. The presence of colic did not predict the development of any of the most common premonitory symptoms (Table 2). Other syndromes were less common (Fig. 4).

**Table 2:** Analysis of association between the most common premonitory symptoms reported and infantile colic

Commonest premonitory symptoms reported	*N*	Presence of infantile colic (*N*)	Odds Ratio	*P* value
Yawning	30	16	0.38 (CI 0.21–0.67)	0.18
Fatigue	62	23	0.73 (CI 0.55–0.97)	0.10
Mood change	55	17	0.96 (CI 0.66–1.4)	0.20
Neck stiffness	33	12	0.70 (CI 0.43–1.32)	0.55
Concentration change	16	2	3.00 (0.73–12.39)	0.15

## Conclusions

Here we report a substantial cohort of patients with migraine premonitory symptoms in order to characterise their phenotype with respect to those seen in adults. The data show a comparable set of symptoms documenting their occurrence from the age of 18 months. While certainly not providing prevalence data broadly, these symptoms can be used as a basis for studying the premonitory phase in larger populations of patients. Moreover, they illustrate the probable fundamental sharing of neurobiological mechanisms between childhood and adolesecent migraine and the condition in adults.

The mean number of premonitory symptoms was three and the highest number of premonitory symptoms recorded was seven. This is comparable to adult studies [7]. It is possible that children may not report symptomatology as frequently as adults, and that when they display symptoms, they are not recognised by parents and carers as being part of the migraine attack [2]. In this cohort, the majority of the children were aged 5 to 12 years, so given this age range there is likely to have been some variability in the reporting of symptomatology. The authors mitigate reporting by probing each patient with these symptoms; this yields a more complete analysis although does impart a small time burden during the consultation. There was no statistically significant association between the reporting of more than two premonitory symptoms with age, sex and headache diagnosis. This is compatible with some other studies, although in the adult based study of Schoonman and colleagues [7], females more frequently reported premonitory symptoms compared to males. Reporting increased with increasing age in another study [2].

The phenotype of symptoms reported here was comparable to adults [5]. Here we did not report less usual premonitory symptoms, such as face changes reported by others from retrospective data [18]. Symptoms, such as hypersalivation and facial pallor, were reported in our patient cohort but these were less common, and we would interpret them as possible cranial autonomic symptoms going forward [22].

Premonitory symptoms, and understanding the basis and neurobiology of this early phase of a migraine attack, may help develop early acute therapies for migraine. Studies have looked at early treatment of attacks with domperidone [23–25] and naratriptan [26]; these studies have been small and the literature is lacking large randomised placebo-controlled trials in this regard. The phenotype of some of the commonly reported premonitory symptoms such as yawning, mood change and sleep disturbance suggest the involvement of brainstem and hypothalamic structures, including dopaminergic nuclei and such nuclei have already been implicated as playing a role in trigeminocervical nociception [22]. Additionally such structures have been shown in a functional imaging study to be activated during the premonitory stage in the absence of pain [20, 27].

### Limitations

This study has important limitations in that all the patients were preselected as having reported premonitory symptomatology at their first Specialist Headache Clinic consultation. We sought not to determine the prevalence of the symptoms in the entire clinic in contrast to developing a better understanding of the range of symptoms when they were present. The use of clinic letters limits exploring the symptomatology in patients, although we (PP/PJG) have been interested in the question of premonitory symptoms over the period of review, which has standardised to a large extent what is recorded. Moreover, we tend to produce clinic letters with an eye to audit in the future to maintain clinical standards that lend themselves to this type of retrospective review. Additionally this patient cohort was clinic based rather than population based, and generalisability of the findings is not clear. Chronic migraine was the most common headache diagnosis in this study likely because we are a teriary headache centre. This may alter reporting of premonitory symptomatology in those children with frequent, daily or continuous headache. The frequency of migraine headache in this cohort has the complexity of the overlap between premonitory and postdromal symptoms. This issue needs further characterisation going forward. We did not determine the prevalance of premonitory symptomatology in our clinic overall nor analyse whether symptoms were able to reliably warn of an impending headache attack. However, we have demonstrated a wider range of premonitory symptoms in the paediatric population across a wide range of age groups. We have also examined the association between the childhood episodic syndomes and the development of premonitory symptoms.

Recognising the presence of the premonitory phase of migraine in children allows education of parents and carers about such symptoms and better recognition of attacks, particularly in younger children. Future studes could provide questionnaries or electronic diaries for these symptoms to be reported by parents and patients and their relationship to attack onset and their ability to predict an attack could be assessed. This would be helpful in ascertaining the true prevalance of premonitory symptoms prospectively in a population based sample, their phenotype and their ability to predict an impending headache in a paediatric and adolescent population. Understanding this would allow randomised controlled trials of treatment in the paediatric population, as well as support further functional imaging and pharmacologcical research against novel therapeutic targets, to aid better treatment of this common and disabling condition.

## References

[1] Abu-Arefeh I, Russell G (1994). Prevalence of headache and migraine in schoolchildren. BMJ (Clinical research ed).

[2] Mortimer MJ, Kay J, Jaron A (1992). Epidemiology of headache and childhood migraine in an urban general practice using Ad Hoc, Vahlquist and IHS criteria. Dev Med Child Neurol.

[3] Headache Classification Committee of the International Headache Society (2013). The International Classification of Headache Disorders, 3rd edition (beta version). Cephalalgia.

[4] Giffin NJ, Lipton RB, Silberstein SD, Olesen J, Goadsby PJ (2016). The migraine postdrome. An electronic diary study. Neurology (Minneap).

[5] Giffin NJ, Ruggiero L, Lipton RB, Silberstein S, Tvedskov JF, Olesen J (2003). Premonitory symptoms in migraine: an electronic diary study. Neurology.

[6] Quintela E, Castillo J, Munoz P, Pascual J (2006). Premonitory and resolution symptoms in migraine: a prospective study in 100 unselected patients. Cephalalgia.

[7] Schoonman GG, Evers DJ, Terwindt GM, van Dijk JG, Ferrari MD (2006). The prevalence of premonitory symptoms in migraine: a questionnaire study in 461 patients. Cephalalgia.

[8] Amery WK, Waelkens J, Vandenbergh V (1986). Migraine warnings. Headache.

[9] Drummond PD, Lance JW (1984). Neurovascular disturbances in headache patients. Clin Exp Neurol.

[10] Isler H-R (1986) Frequency and time course of premonitory phenomena. In: Amery WK, Wauquier A, (eds) The Prelude to the Migraine Attack. Balliere Tindall, London

[11] Rasmussen BK, Olesen J (1992). Migraine with aura and migraine without aura: an epidemiological study. Cephalalgia.

[12] Spiri D, Rinaldi VE, Titomanlio L (2014). Pediatric migraine and episodic syndromes that may be associated with migraine. Ital J Pediatr.

[13] Faria V, Erpeldin N, Lebel A, Johnson A, Wolf R, Fai D (2015). The migraine brain in transition: girls vs boys. Pain.

[14] Gee SJ (1882). On fitful or recurrent vomiting. St Bart’s Hosp Rep.

[15] Mira E, Piacentino G, Lanzi G, Balotti U, Fazzi E (1984). Benign paroxysmal vertigo in childhood: a migraine equivalent. ORL J Otorhinolaryngol Relat Spec.

[16] Giffin NJ, Benton S, Goadsby PJ (2002). Benign paroxysmal torticollis of infancy: 4 new cases and linkage to CACNA1A. Dev Med Child Neurol.

[17] Gelfand AA, Thomas KC, Goadsby PJ (2012). Before the headache: infant colic as an early life expression of migraine. Neurology.

[18] Cuvellier JC, Mars A, Vallee L (2009). The prevalence of premonitory symptoms in paediatric migraine: a questionnaire study in 103 children and adolescents. Cephalalgia.

[19] Schulte LH, Jurgens TP, May A (2015). Photo-, osmo- and phonophobia in the premonitory phase of migraine: mistaking symptoms for triggers?. J Headache Pain.

[20] Maniyar FH, Sprenger T, Monteith T, Schankin C, Goadsby PJ (2014). Brain activations in the premonitory phase of nitroglycerin triggered migraine attacks. Brain.

[21] Goadsby PJ, Lipton RB (1997). A review of paroxysmal hemicranias, SUNCT syndrome and other short-lasting headaches with autonomic features, including new cases. Brain.

[22] Akerman S, Holland P, Goadsby PJ (2011). Diencephalic and brainstem mechanisms in migraine. Nat Rev Neurosci.

[23] Waelkens J (1982). Domperidone in the prevention of complete classical migraine. Br Med J.

[24] Waelkens J (1984). Dopamine blockade with domperidone: bridge between prophylactic and abortive treatment of migraine? A dose-finding study. Cephalalgia.

[25] Waelkens J (1985). Warning symptoms in migraine: characteristics and therapeutic implications. Cephalalgia.

[26] Luciani R, Carter D, Mannix L, Hemphill M, Diamond M, Cady R (2000). Prevention of migraine during prodrome with naratriptan. Cephalalgia.

[27] Maniyar FH, Sprenger T, Monteith T, Schankin CJ, Goadsby PJ (2015). The premonitory phase of migraine- what can we learn from it?. Headache.

